# The Ubiquitin-Specific Protease 18 Promotes Hepatitis C Virus Production by Increasing Viral Infectivity

**DOI:** 10.1155/2019/3124745

**Published:** 2019-11-18

**Authors:** Yujia Li, Max Xuezhong Ma, Bo Qin, Liang-Tzung Lin, Christopher D. Richardson, Jordan Feld, Ian D. McGilvray, Limin Chen

**Affiliations:** ^1^Institute of Blood Transfusion, Chinese Academy of Medical Sciences, Peking Union Medical College, Chengdu, Sichuan 610052, China; ^2^Toronto General Research Institute, University of Toronto, Toronto, Ontario, Canada M5S 1A1; ^3^Department of Infectious Diseases, The 1st Affiliated Hospital of Chongqing Medical University, Chongqing, China; ^4^Department of Microbiology and Immunology, School of Medicine, College of Medicine, Taipei Medical University, Taipei, Taiwan; ^5^Graduate Institute of Medical Sciences, College of Medicine, Taipei Medical University, Taipei, Taiwan; ^6^Department of Microbiology & Immunology, Dalhousie University, Halifax, Nova Scotia, Canada B3H 4R2; ^7^Department of Medicine, University of Toronto, Toronto, Canada M5S 1A1; ^8^Department of Surgery, University of Toronto, Toronto, Canada M5S 1A1

## Abstract

**Background and Aims:**

Ubiquitin-specific protease 18 (USP18) is involved in immunoregulation and response to interferon- (IFN-) based treatment in patients chronically infected with hepatitis C virus (HCV). We investigated whether and how its upregulation alters HCV infection.

**Methods:**

Overexpression of wild-type (USP18 WT) or catalytically inactive mutant (USP18 C64S) USP18 was examined for effects on HCV replication in the absence and presence of IFN*α* or IFN*λ* using both the HCV-infective model and replicon cells. The IFN signaling pathway was assessed via STAT1 phosphorylation (western blot) and downstream ISG expression (real-time PCR). Mechanistic roles were sought by quantifying microRNA-122 levels and J6/JFH1 infectivity of Huh7.5 cells.

**Results:**

We found that overexpression of either USP18 WT or USP18 C64S stimulated HCV production and blunted the anti-HCV effect of IFN*α* and IFN*λ* in the infective model but not in the replicon system. Overexpressed USP18 showed no effect on Jak/STAT signaling nor on microRNA-122 expression. However, USP18 upregulation markedly increased J6/JFH1 infectivity and promoted the expression of the key HCV entry factor CD81 on Huh7.5 cells.

**Conclusions:**

USP18 stimulates HCV production and blunts the effect of both type I and III IFNs by fostering a cellular environment characterized by upregulation of CD81, promoting virus entry and infectivity.

## 1. Introduction

As one of the key effectors in the innate immune system, type I interferon (IFN) plays diverse roles in host defense against virus infection and has been recommended or studied as important/potential therapy in patients with virus infections such as hepatitis B virus (HBV) [1], hepatitis C virus (HCV) [2], hepatitis D virus (HDV) [3], and enterovirus 71 [4]. Unfortunately, type I IFN signaling is a “double-edged sword” [5], because it confers complicated action by regulating the expression of various interferon-stimulated genes (ISGs), which on the one hand control virus replication but on the other hand are involved in persistent viral infection. It is such an important target for which viruses, such as HCV, developed special survival strategies to evade host immune attack and benefit their replication. Thus, investigating how interferon signaling and effector mechanisms are altered in viral infection is critical to understand the intricate host-virus interaction.

Before 2011 when revolutionary direct-acting antivirals (DAAs) were developed, IFN-based therapy remained the most effective treatment for HCV infection. However, it is very challenging to choose an appropriate therapy strategy for every patient because their responses to the IFN-based treatment varied a lot. We have described a dichotomous hepatic gene expression that is linked to treatment response [6, 7]: patients with high expression of a subset of ISGs in hepatocytes were resistant to subsequent treatment with pegylated IFN*α*/ribavirin (PegIFN/Rib), while the patients with much lower expression of hepatic ISGs were very responsive to treatment with PegIFN/Rib. This nonresponder phenotype characterized by discrete patterns of “high ISG” expression has subsequently been confirmed by a number of laboratories [8, 9]. Three of the genes in the hepatocyte-expressed “high-ISG” subset are linked in the same ubiquitin-like biochemical pathway: interferon-stimulated gene 15 (ISG15), cyclin E-binding protein (Herc5/CEB1), and ubiquitin-specific protease 18 (USP18). In this pathway, ISG15 is covalently linked to target proteins by a tightly regulated series of E1/E2/E3 proteins: activating E1 enzyme (Ube1L), conjugating E2 enzyme (UbcH8), and E3 ligase (CEB1, Efp) [10]. ISG15 is cleaved from its targets by the USP18 cysteine protease. The consequences of protein ISGylation (the process of ISG15 conjugation to its target proteins) are currently under investigation, but the process clearly has implications for viral infection in a manner that is specific to the virus in question. ISG15 has antiviral activity for some viruses, such as influenza A and B viruses, herpesviruses, and Sindbis viruses, whereas for HIV, the ISGylation of the HIV gag protein is required for HIV viral egress from the cell [11]. ISGylation may also, in some circumstances, function as a negative regulator of the innate immune response by conjugating to intracellular viral sensor molecules, such as retinoic acid-inducible gene I (RIG-I) to promote viral replication [12].

The ISG15/USP18 pathway is likely to play a role in viral hepatitis, again in a virus-specific manner [13]. Kim et al. found that hepatitis B viral replication was not affected by loss of ISGylation in Ube1L-/- mice [14], while we and others found that ISGylation stimulates HCV replication in vitro [15, 16]. USP18's role in viral hepatitis may reflect an underlying effect in the innate immune response. For example, the CD169+ metallophilic macrophages with upregulated USP18 reduced IFN-induced capture of virus particles, allowing locally restricted replication of vesicular stomatitis virus (VSV) [17]. USP18-knockout mice experienced pronounced increases in protein ISGylation and were resistant to infection by lymphocytic choriomeningitis virus (LCMV), VSV, Sindbis virus, HIV, and other viral infection involving the ISGylation process (reviewed in [18]). We have previously shown that downregulation of USP18 augments the anti-HCV effect of IFN*α* [19]. Originally, we attributed this effect uniquely to an exaggeration of IFN signaling, but the data described above raised the possibility that the role of USP18 in HCV might be more complicated than previously thought.

In this study, we used a human full-length USP18 wild type and an enzymatically inactive mutant to dissect the role of USP18 in the molecular pathogenesis of HCV. Our findings demonstrated that USP18 could create a cellular milieu that favored HCV and stimulated HCV production in a manner that was independent of ISG15.

## 2. Materials and Methods

### 2.1. Cell Line, J6/JFH Culture Model, and Plasmid DNA Transfection

Huh7.5 cells and the HCV infectious clone J6/JFH1, the full-length chimerical genome from the infectious JFH1 (genotype 2a) isolated from a Japanese patient with fulminant hepatitis [20], were kindly provided by Dr. Charles Rice (Rockefeller University, New York). Briefly, the J6/JFH1 RNA transcript was generated and electroporated into Huh7.5 cells. The culture supernatant was collected and applied to naive Huh7.5 cells for viral passage [21]. Because of abolished virus-induced IFN production resulting from RIG-I mutation, Huh7.5 cells can support complete and efficient HCV replication [22]. HCV genotype 1b (Con1b; AB12-A2) and genotype 2a (JFH1; sbJFH1-B2) subgenomic replicon-containing Huh-7 cells were also used [23]. The AB12-A2 cell line is a Huh-7.5 line carrying subgenomic full-length HCV genotype 1b replicon, and the sbJFH1-B2 cell line is a Huh-7 line cell population containing HCV JFH1 RNA. The replicon cell lines were maintained in DMEM supplemented with 10% fetal calf serum (FCS), 100 IU/ml penicillin and streptomycin, 100 *μ*g/ml nonessential amino acid, and 1 mg/ml G418.

Plasmid DNA (prepared using Qiagen Maxiprep kit) transfection was performed with Lipofectamine 2000 as per the manufacturer's protocols (Invitrogen).

### 2.2. USP18 Plasmid Constructs

The human full-length USP18 gene was cloned into pcDNA-DEST53 fused to GFP at the N-terminus (Invitrogen) (USP18 wild type (USP18 WT)). Briefly, human USP18 ORF (in the pENTER221 entry vector, Invitrogen) was cloned into the destination vector (PcDNA-DEST53) by LR recombination. Positive clones were confirmed by sequencing across the junctions. USP18 protein expression was confirmed by western blot (anti-GFP and anti-USP18 antibodies). USP18 mutant forms C64S (referred to as USP18 C64S in the following experiments), C64/65S, and C65S were created by mutating cysteine to serine at point 64, both 64 and 65, or 65 by the GeneTailor site-directed mutagenesis kit (Invitrogen). The primers used were as follows: USP18 forward primer for C64S: 5′-caacattggacagaccAgctgccttaactccttga-3′; USP18 forward primer for C64/65S: 5′-caacattggacagaccAgcAgccttaactccttga-3′; USP18 forward primer for C65S: 5′-caacattggacagacctgcAgccttaactccttga-3′; and USP18 reverse primer for these mutant forms: 5′-ggtctgtccaatgttgtgtaaaccaaccaggccat-3′. After methylation, pENTER221-USP18 was used as a template for PCR reactions using the above mutant primer pairs, and the resulting mutant form of pENTER221-USP18 was screened on LB/agar plates containing 100 *μ*g/ml kanamycin. pcDNA3.1-USP18 was constructed by cloning the wild-type human full-length USP18 gene into pcDNA3.1 (Invitrogen). All positive clones were sequence verified. Blank vectors pcDNA-DEST53 and pcDNA3.1 were used as controls as indicated in the manuscript.

### 2.3. Confirmation of USP18 Protease Activity: ISG15 Cleavage *In Vitro* and *In Vivo*

An ISG15/GST fusion protein construct was created by cloning the ISG15/GST fusion gene into the pcDNA4/HisMax vector (Invitrogen); the sequence was verified. ISG15/GST fusion protein expression in Huh7.5 cells was confirmed by western blot (polyclonal anti-human ISG15 antibody, Cedarlane) 2 days posttransfection. *In vivo* ISG15 cleavage activity of USP18 WT and USP18 mutant forms was analyzed by treating the USP18-transfected cells with IFN*α* (0-100 IU/ml) for 16 hours. ISG15 and ISGylation were detected by western blot as previously described [15], and the band densities were analyzed using ImageJ software.

### 2.4. Quantification of HCV RNA and Infectious HCV Virions

Total intracellular RNA was harvested and purified with 96-well RNA-easy columns as recommended by the manufacturer (Qiagen, Mississauga, ON, Canada). HCV-RNA and glyceraldehyde-3-phosphate dehydrogenase (GAPDH) RNA were quantified by real-time PCR (SYBR Green, Qiagen) using an ABI Prism 7700 sequence detector (Applied Biosystems, Foster City, CA), and the results were analyzed with SDS 1.9 software from Applied Biosystems as described previously [15, 19]. Primers are listed in Table 1.

Virus titers were determined by limiting dilution analysis as described previously [24]. HCV-positive wells were counted, and the 50% infectious dose was calculated by the method of Reed and Muench [25].

### 2.5. Assessment of Jak/STAT Signaling and microRNA-122 Expression

Huh7.5 cells were seeded at 3 × 10^5^/ml, 2 ml per well in 6-well plates in antibiotic-free medium for 24 hours before either 4 *μ*g USP18 WT or 4 *μ*g USP18 mutant form (USP18 C64S) was transfected into each well. 36 hours posttransfection, 10 U/ml IFN*α* was added to each well. The cells were harvested at 0 min, 30 min, 2 hours, 4 hours, 8 hours, and 24 hours posttreatment. Total protein was extracted using lysis buffer and 1 mM EDTA with protease inhibitor cocktail (Sigma). Phospho-STAT1 (Tyr701) and total STAT1 were detected by western blot (Cell Signaling, USA), and the band densities were analyzed using ImageJ software. At each time point, total RNA was extracted by TRIzol (Thermo Fisher Scientific, USA), and ISG mRNAs were determined by real-time PCR described above with the primers listed in Table 1. All the primers were self-designed with the Primer3 program and were synthesized by a commercial company.

MicroRNA-122 expression levels were also determined using the microRNA-122 kit (Applied Biosystems, USA) following the manufacturer's protocols and normalized to U6.

### 2.6. CD81 Expression Quantification

The CD81 expression on USP18 overexpressed cells and control cells was quantified by using the flow cytometry technique with specific antibodies. Briefly, the cells (1 × 10^6^) for each acquisition of samples were washed twice in fluorescence-activated cell sorting (FACS) buffer (phosphate-buffered saline (PBS, pH 7.4) containing 2% FCS and 0.02% NaN3) and resuspended in 100 *μ*l of FACS buffer and then stained with a PE-conjugated mouse monoclonal antibody against human CD81 (Santa Cruz Biotechnology) chilled on ice for 40 minutes. The cells were washed twice in FACS buffer and prepared with FIX & PERM reagents (Invitrogen Life Technologies) following the manufacturer's protocol, then stained with either a primary mouse monoclonal antibody against Flag (Invitrogen Life Technologies®) or a rabbit polyclonal antibody against USP18 incubated with the relevant antibody or relevant isotype matched control antibodies at 4°C for 40 minutes. The cells were washed twice in FACS buffer and resuspended in 100 *μ*l of FCS for 30 minutes to prevent nonspecific antibody binding. This was followed by three washes in FACS buffer and incubation for 30 minutes at 4°C with the secondary goat anti-mouse IgG FITC (Santa Cruz Biotechnology) or goat anti-rabbit IgG Alexa 488. The cells were washed thrice and analyzed using the FACS Calibur flow cytometer (Becton Dickinson Immunocytometry Systems). Live cells were gated on the basis of forward and side scatter, and a minimum of 10,000 events were analyzed. FlowJo software (FlowJo, USA) was used to perform the data analysis.

### 2.7. Statistics

All the experiments were repeated at least three times, and where appropriate, Student's *t*-test was used to compare categorical values. *p* < 0.05 was considered statistically significant.

## 3. Results

### 3.1. Expression of Human Full-Length USP18 and Catalytic Activity of USP18 WT and USP18 C64S Forms in Huh7.5 Cells

**Figure 1:**
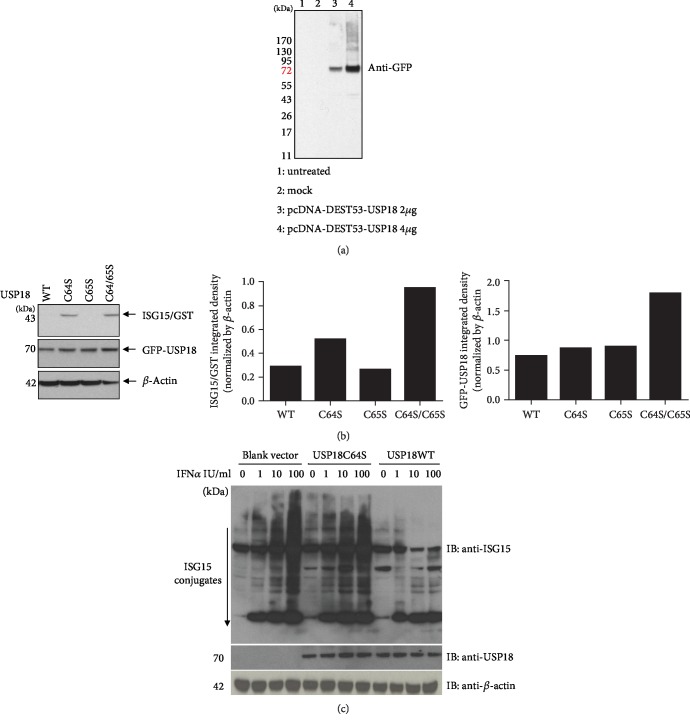
Human USP18 expression and catalytic activity in Huh7.5 cells. (a) Huh7.5 cells were seeded at 3 × 10^5^/ml, 2 ml per well in 6-well plates in antibiotic-free medium for 24 hours before 4 *μ*g empty vector pcDNA-DEST53, 2 *μ*g USP18 WT, or 4 *μ*g USP18 WT was transfected into each well. 48 hours posttransfection, total protein was extracted to detect GFP tag by western blot. (b) Cleavage of ISG15-GST fusion in vitro. Huh7.5 cells were transfected with various USP18 plasmids (wild-type USP18, WT; USP18 C64S, C64S; USP18 C65S, C65S; or USP18 C64/65S, C64/65S) in combination with ISG15-GST. 48 hours posttransfection, total protein was extracted to detect ISG15 and USP18 by western blot. (c) Cleavage of ISG15 conjugates in IFN*α*-treated Huh7.5 cells. Huh7.5 cells were transfected with an empty vector (vector), USP18 WT, or USP18 C64S. 24 hrs later, the cells were treated with IFN*α* (0-100 U/ml) for 24 hours, after which western bot was performed to analyze expressions of ISG15 conjugates and USP18. Untreated: untreated control; mock: transfected with 4 *μ*g empty vector pcDNA-DEST53; pcDNA-DEST53-USP18: transfected with wild-type USP18 (USP18 WT).

Wild-type USP18-GFP fusion protein was expressed in Huh7.5 cells with an appropriate dose-response pattern (Figure 1(a)), and the transfection efficiency was shown by the GFP expression in the cells (Supplement [Supplementary-material supplementary-material-1]). USP18 is a cysteine protease, and cysteine 61 (C61) has been reported to be essential for its ability to cleave ISG15 from target proteins in murine cells [26]. There are two cysteine residues near this location in the human USP18 protein, at positions 64 and 65. In order to confirm which Cys is essential for the protease activity of human USP18, three different mutants were constructed: mutating C64 alone, both C64 and C65, or only C65 to serine. We then expressed an ISG15/GST fusion protein in the presence or absence of the various USP18 constructs. In these experiments, expression of wild-type USP18 led to the release of the ISG15 protein; USP18 C64S and C64/C65 mutants did not have this ability, while the C65S mutant did (Figure 1(b)). Thus, in our model system, C64 but not C65 of human USP18 is critical for USP18 protease activity. In order to test the ISG15 protease activity of USP18 WT and USP18 C64S under more physiologically relevant conditions, these constructs were overexpressed prior to exposing the cells to different amount of IFN*α* stimulation. Increased USP18 cleavage activity was observed in IFN*α*-stimulated cells (up to 100 IU/ml) as shown by decreased ISG15 conjugates (Figure 1(c)). As expected, USP18 C64S, the enzymatically inactive mutant form of USP18, did not have this effect.

### 3.2. USP18 Stimulates HCV Production and Blunts Anti-HCV Activity Induced by IFN*α* or IFN*λ*

**Figure 2:**
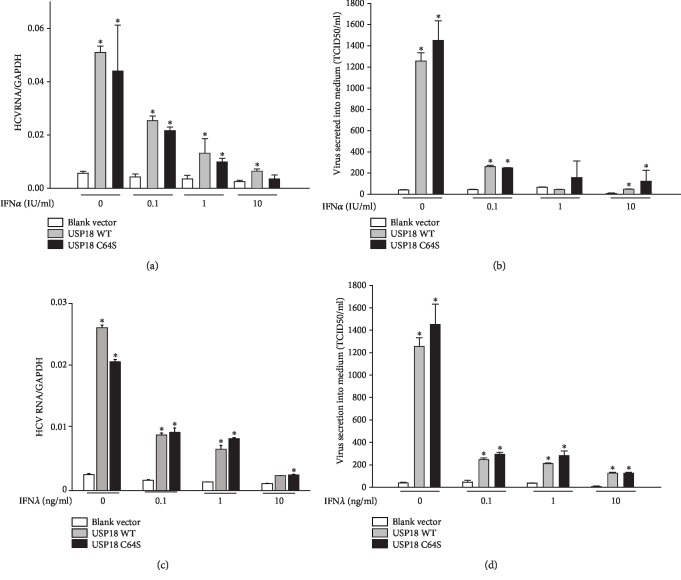
Protease-independent promotion of HCV production and blunting of type I and type III IFN anti-HCV activity by USP18. Huh7.5 cells were seeded at 1.5 × 10^5^/well in 12-well plates one day before 2 *μ*g blank vector pcDNA-DEST53, 2 *μ*g USP18 WT, or 2 *μ*g USP18 C64S was transfected. 48 hours posttransfection, J6/JFH1 virus was added (MOI = 4) and incubated for 4 hours before the culture medium was removed. And then, the cells were washed and supplied with fresh medium and cultured for another 48 hours. The intracellular total RNA and the culture medium were collected. J6/JFH1 RNA (a) and J6/JFH1 virion production (b, c) in the presence and absence of IFN*α* or IFN*λ* were detected by real-time PCR or limiting dilution analysis, respectively. Results are presented as means ± SD (*n* ≥ 3). ^∗^*p* < 0.05.

We next asked whether the USP18 protein and its protease activity directly affect HCV production in the presence and absence of IFN*α* or IFN*λ*. In the absence of IFN*α*, overexpression of either USP18 WT or USP18 C64S increased HCV RNA (Figure 2(a)) and HCV virion titers (Figure 2(b)) by 10-25-fold, respectively. In the presence of IFN*α*, overexpression of USP18 WT blunted IFN*α* anti-HCV activity (Figures 2(a) and 2(b)). Taken together, these data demonstrate that USP18 can promote HCV production and modulate IFN*α* anti-HCV activity independent of its ISG15 protease activity in the J6/JFH1 HCV infectious culture system.

IFN*λ* is a member of the relatively new type III IFN family [27], which signals through a different receptor other than the type I IFNs. The effects of USP18 on type I IFN signaling may in part be mediated via binding of USP18 to IFNAR2 (type 1 IFN receptor subunit 2, part of the type I IFN receptor) [28]. Although USP18 deficiency resulted in hypersensitivity of mouse mammary epithelial cells to IFN*λ* which could be restored by USP18 overexpression [29], the study [30] in human cells demonstrated that IFN-induced USP18 expression specifically suppresses the response to IFN*α*, but not to IFN*β* or IFN*λ*. The contradictory findings indicated that USP18 might regulate IFN signaling in various pathways depending on the cell type. Thus, we also investigated whether the anti-HCV effect of IFN was influenced by USP18 or not. In the present cell model, overexpression of either USP18 WT or USP18 C64S blunted the IFN*λ* anti-HCV activity as shown by the upregulated intracellular HCV RNA (Figure 2(c)) and increased HCV virion secretion into medium (Figure 2(d)). These data suggest that the pro-HCV production and blunting effect of USP18 are not dependent on specific IFNs.

### 3.3. Overexpression of USP18 Has Little Effect on Type I IFN Jak/STAT Signaling

**Figure 3:**
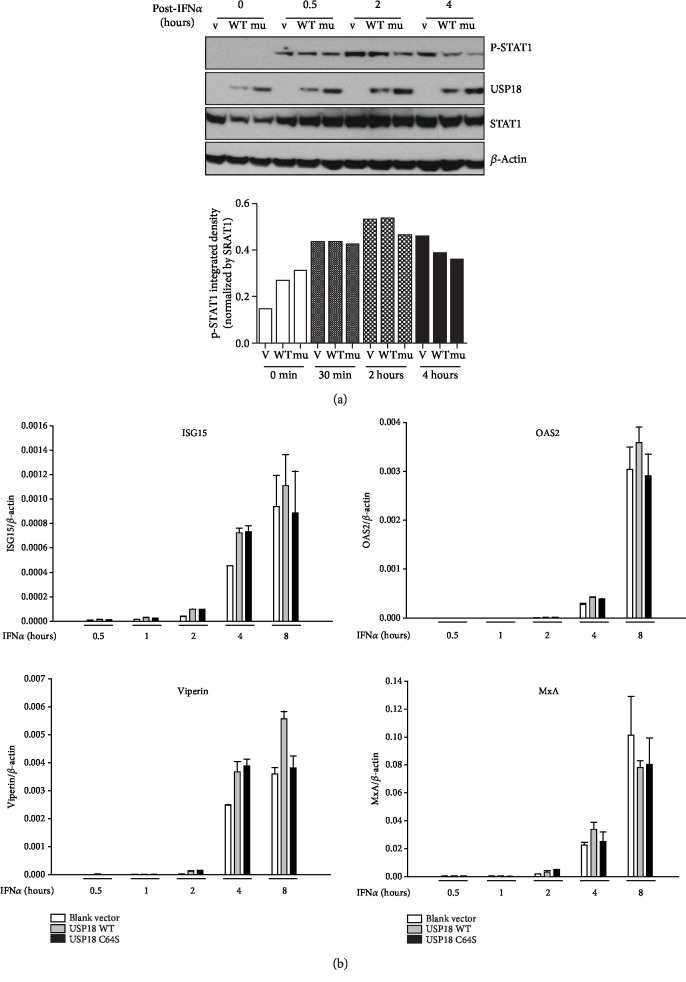
USP18 has no inhibitory effect on Jak/STAT signaling in Huh7.5 cells. Huh7.5 cells were seeded at 3 × 10^5^/ml, 2 ml per well in 6-well plates in antibiotic-free medium for 24 hours before 4 *μ*g blank vector pcDNA-DEST53, 4 *μ*g USP18 WT, or 4 *μ*g USP18 C64S was transfected into each well. 36 hours posttransfection, 100 IU/ml IFN*α* was added to each well. The cells were harvested at 0 min, 30 min, 2 hours, 4 hours, and 8 hours posttreatment. Total protein was extracted to detect USP18, phospho-STAT1 (Tyr701), and total STAT1 by western blot (a, upper), and phospho-STAT1 integrated density was normalized by STAT1 (a, bottom). Total RNA was extracted to detect ISG mRNAs by real-time PCR (b). V: transfected with 4 *μ*g blank vector pcDNA-DEST53; WT: transfected with 4 *μ*g USP18 WT; mu: transfected with 4 *μ*g USP18 C64S. Results are presented as means ± SD (*n* ≥ 3).

As noted above, a previous study reported that murine USP18 expressed in human cells could block the Jak1-IFNAR2 interaction independent of its protease activity [28]. We then asked whether IFN*α* signaling was altered in the presence of USP18 overexpression in our model system. We considered upstream STAT1 activation (phosphorylated STAT1 levels), downstream ISG mRNA expression, and STAT1 protein levels. Overexpression of either USP18 WT or USP18 C64S had little effect on STAT1 phosphorylation at early time points (30 min) following IFN*α* treatment but did slightly decrease STAT1 phosphorylation at later time points (2 hours and 4 hours, Figure 3(a), upper). However, when p-STAT1 was normalized to STAT1 (p-STAT1/STAT1), no statistically difference was observed (Figure 3(a), bottom). Consistent with this, the Jak/STAT signaling pathway was not altered as shown by the similar levels of downstream ISG mRNA expression (Figure 3(b)). These data suggest that the pro-HCV production activity of USP18 is not mediated through changes in Jak/STAT signaling.

### 3.4. USP18 Overexpression Has No Effect on HCV Replicon

**Figure 4:**
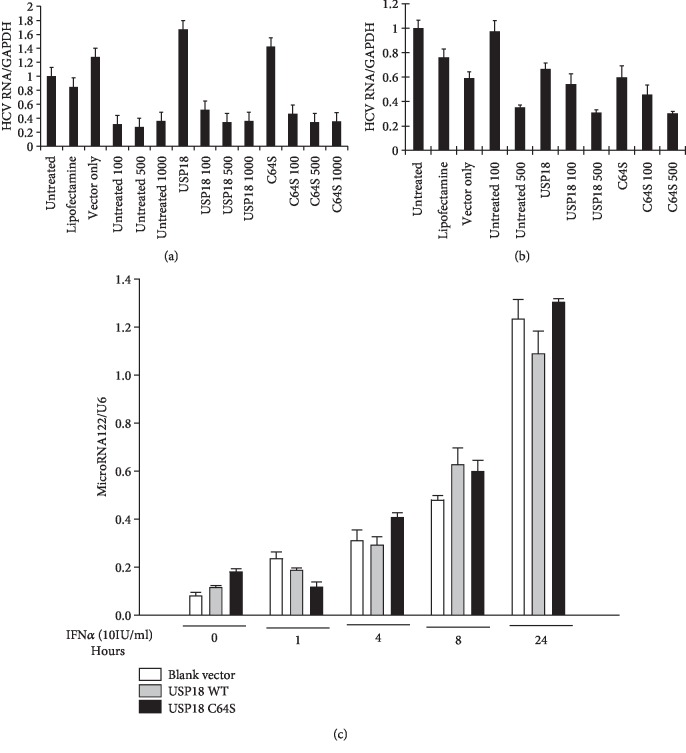
USP18 overexpression does not affect HCV replicons nor microRNA122 expression. 5 × 10^5^ AB12-A2 or sbJFH1-B2 cells were seeded overnight (6-well plates) before transfection with Lipofectamine 2000 in OptiMem with 4 *μ*g USP18 WT or USP18 C64S plasmid DNA. 24 hours posttransfection, protein was collected (RIPA buffer) or cells were treated with IFN*α* (Recombinant, Sigma) at 1, 100, 500, or 1000 IU/ml in DMEM without antibiotics and incubated for 72 h before RNA isolation. Total RNA was collected and subjected to real-time PCR assessment using primers specific for HCV Con1b (a) or HCV JFH1 5′ UTR (b). (c) Huh7.5 cells were transfected with 4 *μ*g blank vector pcDNA-DEST53, 4 *μ*g USP18 WT, or 4 *μ*g USP18 C64S for 48 hours before treatment with IFN*α* (10 IU/ml). MicroRNA 122 levels were determined by a kit and normalized to U6 at different time points indicated post-IFN*α* treatment. Results are presented as means ± SD (*n* ≥ 3).

If the stimulatory effect of USP18 on HCV production was mediated at the level of viral replication, then HCV replication in a noninfectious model (replicon system) should also be affected by USP18 overexpression. In addition, we might also predict that expression of cofactors necessary for HCV RNA replication, such as microRNA-122, which is directly interacting with HCV RNA [31], might be sensitive to the manipulation of USP18. Surprisingly, overexpression of both USP18 WT or USP18 C64S had no effect on HCV RNA replication in either genotype 1b replicon (Con1b; AB12-A2, Figure 4(a)) or genotype 2a subgenomic replicon (JFH1; sbJFH1-B2, Figure 4(b)) cells in the presence or absence of IFN*α*. Moreover, USP18 overexpression also had no effect on microRNA-122 levels (Figure 4(c)). These data argue that USP18 does not directly affect intracellular HCV replication.

### 3.5. Overexpression of USP18 Increases HCV Infectivity in Huh7.5 Cells

**Figure 5:**
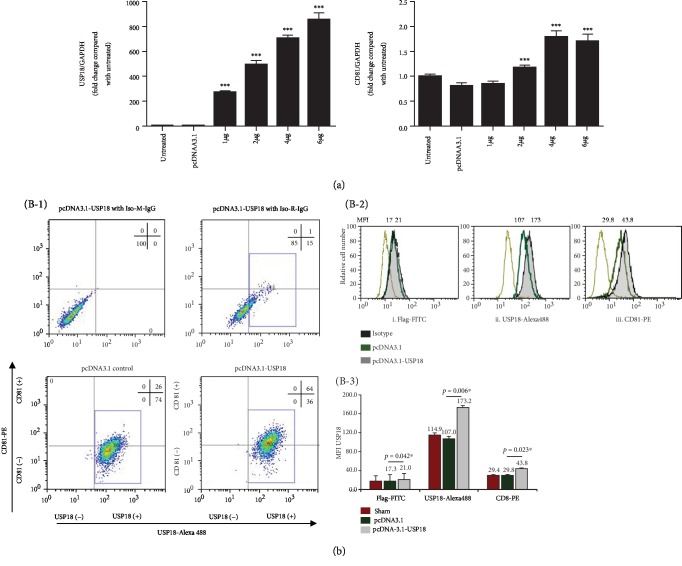
USP18 promotes HCV infectivity and CD81 expression in Huh7.5 cells. (a) Huh7.5 cells were seeded at 3 × 10^5^/ml, 2 ml per well in 6-well plates in antibiotic-free medium for 24 hours before 1 *μ*g, 2 *μ*g, 4 *μ*g, and 6 *μ*g pcDNA3.1-USP18 or 4 *μ*g empty vector pcDNA3.1 was transfected into each well. 48 hours posttransfection, total RNA was extracted; USP18 and CD81 expression was quantified by qRT-PCR and normalized to GAPDH expression as previously described. (b) Huh7.5 cells were seeded at 3 × 10^5^/ml, 2 ml per well in 6-well plates in antibiotic-free medium for 24 hours before 4 *μ*g pcDNA3.1-USP18 was transfected into each well. (B-1) Lower-left and lower right quadrants indicate that the cells were transfected with empty vector pcDNA3.1 or pcDNA3.1-USP18 at 72 hours, with the percentage of cells in respective quadrants. All dot blots show CD81 expression (vertical axis) according to USP18 expression (horizontal axis). Upper-left and right quadrants show the range of isotype control for PE- and Alexa 488-conjugated antibodies. (B-2) Huh7.5 cells with transfected empty vector pcDNA3.1 (open histogram, dark green line) or pcDNA3.1-USP18 DNA (filled histogram) were stained with a mouse anti-flag primary/FITC-conjugated goat anti-mouse secondary antibody, rabbit anti-human USP18 primary/Alexa 488-conjugated goat anti-rabbit secondary antibody, and PE-conjugated anti-human CD81 monoclonal antibody. The cells transfected with pcDNA3.1-USP18 were also stained with mouse and rabbit IgG and isotype control antibodies (open histogram, brown line). (B-3) Comparison of USP18/CD81 expression in or on Huh7.5 cells with sham control, transfected pcDNA3.1 control, and pcDNA3.1-USP18 DNA. Bars indicate MFI of USP18 (FITC and Alexa 488)/CD81 (PE) expression ± STDV. Untreated: untreated control; pcDNA3.1: transfected with 4 *μ*g empty vector pcDNA3.1; 1 *μ*g, 2 *μ*g, 4 *μ*g, and 6 *μ*g: transfected with 1 *μ*g, 2 *μ*g, 4 *μ*g, or 6 *μ*g pcDNA3.1-USP18; sham control: untreated Huh7.5 cells only. Results are presented as means ± SD (*n* ≥ 3). ^∗^*p* < 0.05; ^∗∗^*p* < 0.01; ^∗∗∗^*p* < 0.001.

If USP18 does not contribute to HCV RNA replication but does promote HCV production, it must alter the cellular milieu in a manner that favors the HCV life cycle at steps other than RNA replication. One possibility is that USP18 increases the susceptibility of the cell to infection by HCV. To examine this possibility, we asked whether USP18 overexpression alters HCV infectivity. As shown in Figure 2, increased USP18 expression led to markedly increased HCV infection of Huh7.5 cells by 5-6-fold. Like most other viruses, the infectivity of HCV is mainly determined by the interaction between the viral glycoproteins and a series of attachment factors and entry factors which are involved in the initiation of infection [32]. Therefore, we analyze the expression of the entry factor CD81, which is in the most essential position of the HCV entry factor complex, in Huh7.5 cells. As expected, there was a USP18 concentration-dependent upregulation in CD81 mRNA expression (Figure 5(a)). FACS also revealed a significant upregulation of CD81 expression in parallel with elevated USP18 level (Figure 5(b)).

## 4. Discussion

USP18 is a cysteine protease with specific ISG15 cleavage (deconjugating) activity [33]. Originally cloned from leukemia fusion protein AML1-ETO-expressing mice [34], USP18 is an ISG whose expression level is inducible by type I IFN and is degraded by proteolysis through the SCFSkp2 ubiquitin ligase [35]. The roles of the USP18 pathway in viral infection differ depending on the virus involved and can be mediated through multiple different routes. Although originally attributed to the effects of ISGylation, subsequently it has been shown that USP18 has ISG15-independent effects. Although Usp18-/- mice exhibited less replication of LCMV and VSV, ISG15- or Ube1L-knockout mice had the same sensitivity to LCMV and VSV infection as wild-type mice, indicating that USP18 may behave in an ISG15-independent manner [36]. Specifically, murine USP18 can bind to human IFNAR2 and block type I IFN signaling by competitively interfering with Jak1 binding to the receptor [28]. Thus, at least some of its effects may be mediated through its effects on IFN signaling and not through ISG15 directly. And USP18 also has the ability to affect cellular pathways (and expression of surface proteins), as demonstrated by its ability to (1) regulate the expression of the EGF receptor in carcinoma cells [37, 38], (2) inhibit tumor necrosis factor- (TNF-) related apoptosis-inducing ligand- (TRAIL-) induced apoptosis [39], and (3) employ USP20 to promote deubiquitination of the mitochondrial adaptor protein STING [40]. Taken together, these data illustrate that the effects of USP18 in any given viral infection can be mediated by a number of routes and may or may not be dependent on the USP18 deconjugase function or on a direct inhibition of IFN signaling.

The results of our study suggested that the deISGylation (cleaving ISG15 from its target proteins) process was not involved in the effect of USP18 on HCV. We synthesized a mutant USP18 with no ability to cleave ISG15 from an ISG15/GFP fusion protein. Overexpression of this mutant form of USP18 led to a similar degree of increased HCV production and blunting of the anti-HCV effect of IFN*α*. Furthermore, the work from our laboratory has shown that ISGylation is required for efficient HCV production, in that inhibition of ISGylation by knockdown of the E1 Ube1L enzyme reduces HCV viral titers and RNA [15]. If the effect of USP18 on HCV production were dependent on ISG15 and ISGylation, the decrease in ISGylation seen with the overexpression of USP18 would be expected to inhibit HCV production. In fact, despite a decrease in cellular ISGylation, there is a consistent increase in HCV titers and RNA following the overexpression of wild-type USP18. These data are consistent with an ISG15-independent ability of USP18 to stimulate HCV production.

In earlier work, we found that knockdown of USP18 enhanced the anti-HCV effect of IFN*α*, in concert with increased cellular protein ISGylation and increased activation of the Jak/STAT signaling pathway [19]. It has also been reported that exposure of HLLR1-1.4 cells or primary hepatocytes to either type I or type III IFNs interfered with the cells' ability to further respond to IFN*α* subtypes (desensitization), but that the response to IFN*β* or IFN*λ* is not affected [30]. Although no mechanistic details were given, the authors speculated on a threshold effect linking USP18 expression and type I IFN receptor inhibition. The article figured out that IFN treatment-induced USP18 was sufficient to induce a “refractory” state to IFN*α* because IFN*α* has lower affinity for the receptor compared with IFN*β*. By contrast, in the current study, we found that overexpression of USP18 had little to no effect on IFN*α*-induced ISG expression, only a mild effect on IFN*α* Jak/STAT signaling, and no effect on IFN*α*-induced STAT1 protein expression and yet markedly increased HCV production in the absence of IFN*α* and blunted the anti-HCV effect of IFN*α*. The fact that IFN signaling was enhanced when USP18 expression had been decreased and showed little change when USP18 expression had been increased was more in keeping with its effect on IFN signaling being mediated through an intermediary than through direct binding to the receptor of type I IFN. We did ask whether steric effects from the relatively large GFP protein tagged to the USP18 construct could interfere with binding to the IFN receptor. But similar results were obtained (data not shown) when we created a separate USP18 expression construct with a much smaller His-tag (pDEST26). These data, taken together, argue that binding of USP18 to the IFNAR2 receptor cannot fully explain the effect of USP18 in the present infectious HCV model system. Furthermore, since USP18 is involved in regulating various signaling pathways including the IFN*λ* pathway [18], new evidence will be needed to elucidate whether USP18 could promote HCV production by inhibiting the signaling of type III IFN.

From a mechanistic standpoint, the positive effect of USP18 on HCV production is not mediated at the level of RNA replication *per se*. Overexpression of either USP18 WT or protease-inactive USP18 C64S had no effect in HCV model systems that require only RNA replication (HCV replicon-containing cells). Neither the wild type nor the protease-inactive variant of USP18 altered HCV RNA in two distinct replicon systems. There was also no change in levels of cellular cofactors that have been shown to be important for HCV replication, such as microRNA-122.

Although USP18 upregulation does not seem to influence HCV RNA replication, increased expression of wild-type or mutant USP18 stimulates HCV infectivity in Huh7.5 cells. We have further demonstrated that USP18 upregulation leads to increased surface expression of CD81 which could form a receptor complex for HCV internalization into hepatocytes with other proteins such as calpain-5 (CAPN5) and the ubiquitin ligase casitas B-lineage lymphoma proto-oncogene B (CBLB) [41]. This elevated CD81 expression might be responsible for the increased HCV infectivity and subsequent viral production in Huh7.5 cells. Thus, our work pointed out that the role of USP18 in innate immunity and in particular in a cell's susceptibility and response to viral infection is considerably more complex than previously thought although the precise mechanism for this effect remains to be investigated.

In conclusion, our present study demonstrates that USP18 contributes to the viral/host interplay, creating a hepatocellular environment that is more favorable to viral production. The fact that USP18 expression augments markedly increased CD81 expression and HCV infectivity provided some mechanistic insight into the effect and may illustrate a novel means by which HCV can subvert the host innate immune response to its benefit. USP18 is an important modulator of the host innate response and clearly plays an important role in clinical HCV. Further studies investigating the effects of USP18 on other viral infections should be done to reveal its potentiality as a biomarker of diseases and as a therapeutic target.

## Figures and Tables

**Table 1:** Primers used for real-time PCR.

Gene	Full name	Forward primer	Reverse primer
HCV Con1b	Hepatitis C virus Con1b	GCAGAAAGCGTCTAGCCAT	CTCGCAAGCACCCTATCAG
HCV JFH1	Hepatitis C virus JFH1	GCAGAAAGCGCCTAGCCAT	CTCGCAAGCGCCCTATCAG
GAPDH	Glyceraldehyde-3-phosphate dehydrogenase	GCCTCCTGCACCACCAACTG	ACGCCTGCTTCACCACCTTC
ISG15	Interferon-stimulated protein 15	CGCAGATCACCCAGAAGATT	GCCCTTGTTATTCCTCACCA
OAS2	2′,5′-Oligo adenylate synthetase 2	TCAGCGAGGCCAGTAATCTT	GCAGGACATTCCAAGATGGT
Viperin	Viperin	CTTTTGCTGGGAAGCTCTTG	CAGCTGCTGCTTTCTCCTCT
MxA	Myxovirus (influenza virus) resistance 1	GTGCATTGCAGAAGGTCAGA	CTGGTGATAGGCCATCAGGT
*β*-Actin	Beta-actin	CTCCATCCTGGCCTCGCTGT	GCTGTCACCTTCACCGTTCC

## Data Availability

All the data used to support the findings of this study are included in the article.

## Abbreviations

IFN: Interferon
HCV: Hepatitis C virus
ISG: Interferon-stimulated gene
ISG15: Interferon-stimulated gene 15
USP18: Ubiquitin-specific protease 18
Jak/STAT: Janus kinase/signal transducer and activator of transcription
pegIFN/rib: Pegylated interferon/ribavirin
FACS: Fluorescence-activated cell sorting
GAPDH: Glyceraldehyde-3-phosphate dehydrogenase.

## Supplementary Materials

Supplementary Materials Supplemental materials and methods: transfection efficiency evaluation pmaxGFP (Lonza, Switzerland) was used to evaluate the transfection efficiency. Huh7.5 cells were seeded at 3 × 10^5^/ml, 2 ml per well in 6-well plates in antibiotic-free medium for 24 hours before 1 *μ*g, 2 *μ*g, and 4 *μ*g (C) GFP plasmid DNA or 4 *μ*g empty vector (D) was transfected into each well. Fluorescent microscopy images were taken 48 hours post-transfection. An anti-CD81 antibody ([M38], Abcam) was used to block CD81. Briefly, Huh7.5 cells were preincubated for one hour with 1 *μ*g/ml or 20 *μ*g/ml anti-CD81 ([M38], Abcam). The cells were washed 3 times with PBS before the J6/JFH1 virus was added (MOI = 4). After 4 hours of incubation, the cells were washed and left 24 hours before collecting total intracellular RNA. J6/JFH RNA was determined by real-time PCR. Effect of USP18 on other entry or attachment factors in HCV infection. Huh7.5 cells were seeded at 3 × 10^5^/ml, 2 ml per well in 6-well plates in antibiotic-free medium for 24 hours before 1 *μ*g, 2 *μ*g, 4 *μ*g, and 6 *μ*g USP18 WT or 4 *μ*g empty vector pcDNA-DEST53 was transfected into each well. 48 hours posttransfection, total RNA was extracted; SR-BI, occludin, and claudin-1 were quantified by real-time PCR. The primers (purchased from Sangon Biotech, China) are as follows: CD81, forward primer: 5′-GTGATCCTGGGTGCCCT-3′ and reverse primer: 5′-CATCATCCACCACGGCCTGC-3′; SR-BI, forward primer: 5′-ACGACACCGTGTCCTTCCTCG-3′ and reverse primer: 5′-CGGGCTGTAGAACTCCAGCGA-3′; occludin, forward primer: 5′-AGTGTGATAATAGTGAGTGCTATCC-3′ and reverse primer: 5′-TGTCATACCTGTCCATCTTTCTTC-3′; and claudin-1, forward primer: 5′-TTCTCGCCTTCCTGGGATG-3′ and reverse primer: 5′-CTTGAACGATTCTATTGCCATACC-3′.
