# Supplementary material for: The Ubiquitin-Specific Protease 18 Promotes Hepatitis C Virus Production by Increasing Viral Infectivity
Source: Mediators Inflamm. 2019 Nov 18;2019:3124745. doi: 10.1155/2019/3124745 (PMC6906844; doi:10.1155/2019/3124745)
Supplement: Supplementary Materials — Supplemental materials and methods: transfection efficiency evaluation pmaxGFP (Lonza, Switzerland) was used to evaluate the transfection efficiency. Huh7.5 cells were seeded at 3 × 10^5^/ml, 2 ml per well in 6-well plates in antibiotic-free medium for 24 hours before 1 μg, 2 μg, and 4 μg (C) GFP plasmid DNA or 4 μg empty vector (D) was transfected into each well. Fluorescent microscopy images were taken 48 hours post-transfection. An anti-CD81 antibody ([M38], Abcam) was used to block CD81. Briefly, Huh7.5 cells were preincubated for one hour with 1 μg/ml or 20 μg/ml anti-CD81 ([M38], Abcam). The cells were washed 3 times with PBS before the J6/JFH1 virus was added (MOI = 4). After 4 hours of incubation, the cells were washed and left 24 hours before collecting total intracellular RNA. J6/JFH RNA was determined by real-time PCR. Effect of USP18 on other entry or attachment factors in HCV infection. Huh7.5 cells were seeded at 3 × 10^5^/ml, 2 ml per well in 6-well plates in antibiotic-free medium for 24 hours before 1 μg, 2 μg, 4 μg, and 6 μg USP18 WT or 4 μg empty vector pcDNA-DEST53 was transfected into each well. 48 hours posttransfection, total RNA was extracted; SR-BI, occludin, and claudin-1 were quantified by real-time PCR. The primers (purchased from Sangon Biotech, China) are as follows: CD81, forward primer: 5′-GTGATCCTGGGTGCCCT-3′ and reverse primer: 5′-CATCATCCACCACGGCCTGC-3′; SR-BI, forward primer: 5′-ACGACACCGTGTCCTTCCTCG-3′ and reverse primer: 5′-CGGGCTGTAGAACTCCAGCGA-3′; occludin, forward primer: 5′-AGTGTGATAATAGTGAGTGCTATCC-3′ and reverse primer: 5′-TGTCATACCTGTCCATCTTTCTTC-3′; and claudin-1, forward primer: 5′-TTCTCGCCTTCCTGGGATG-3′ and reverse primer: 5′-CTTGAACGATTCTATTGCCATACC-3′. [file 3124745.f1.doc]

**Supplement Figure 1**

**
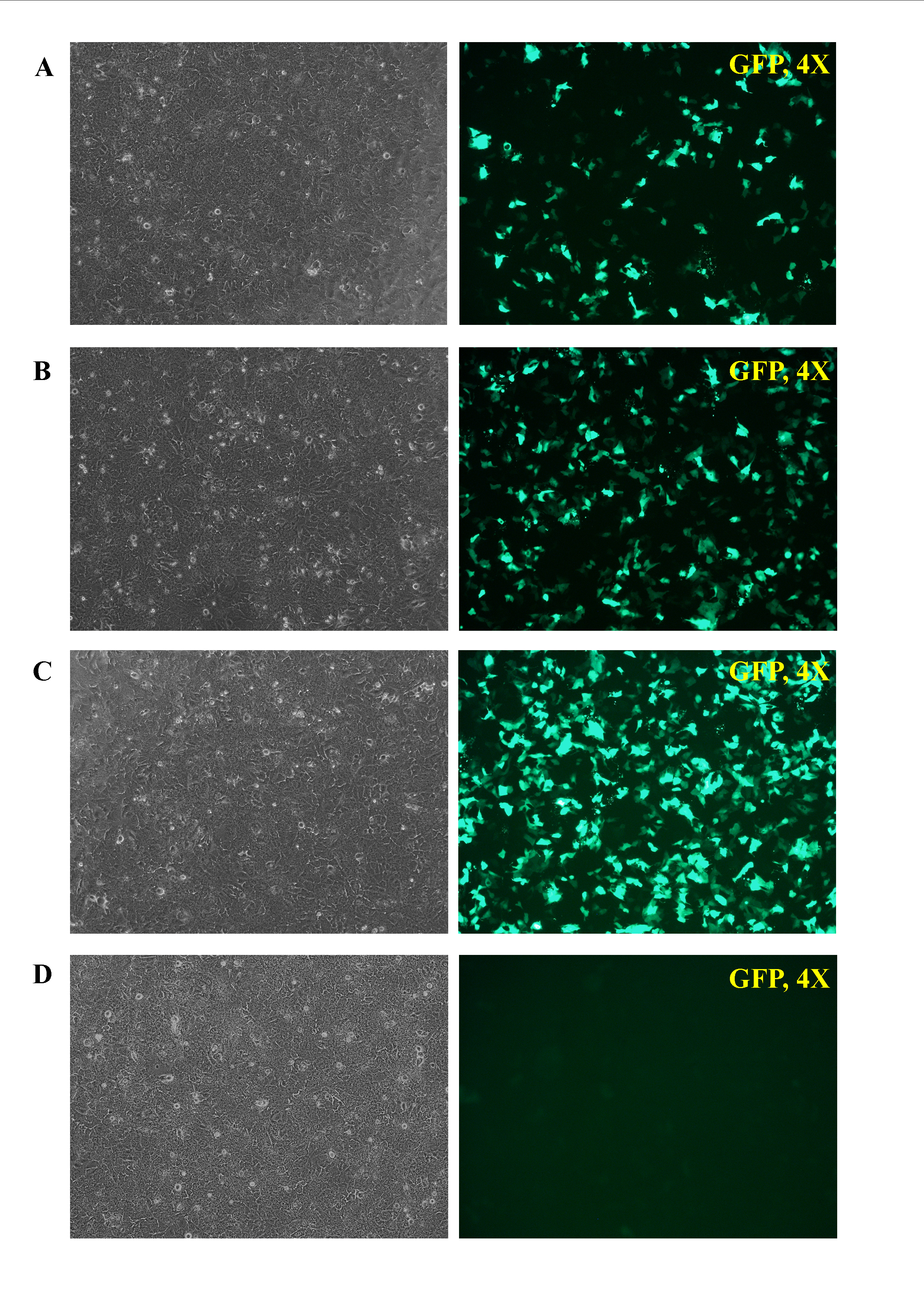
**

**Supplement Figure 2**

**
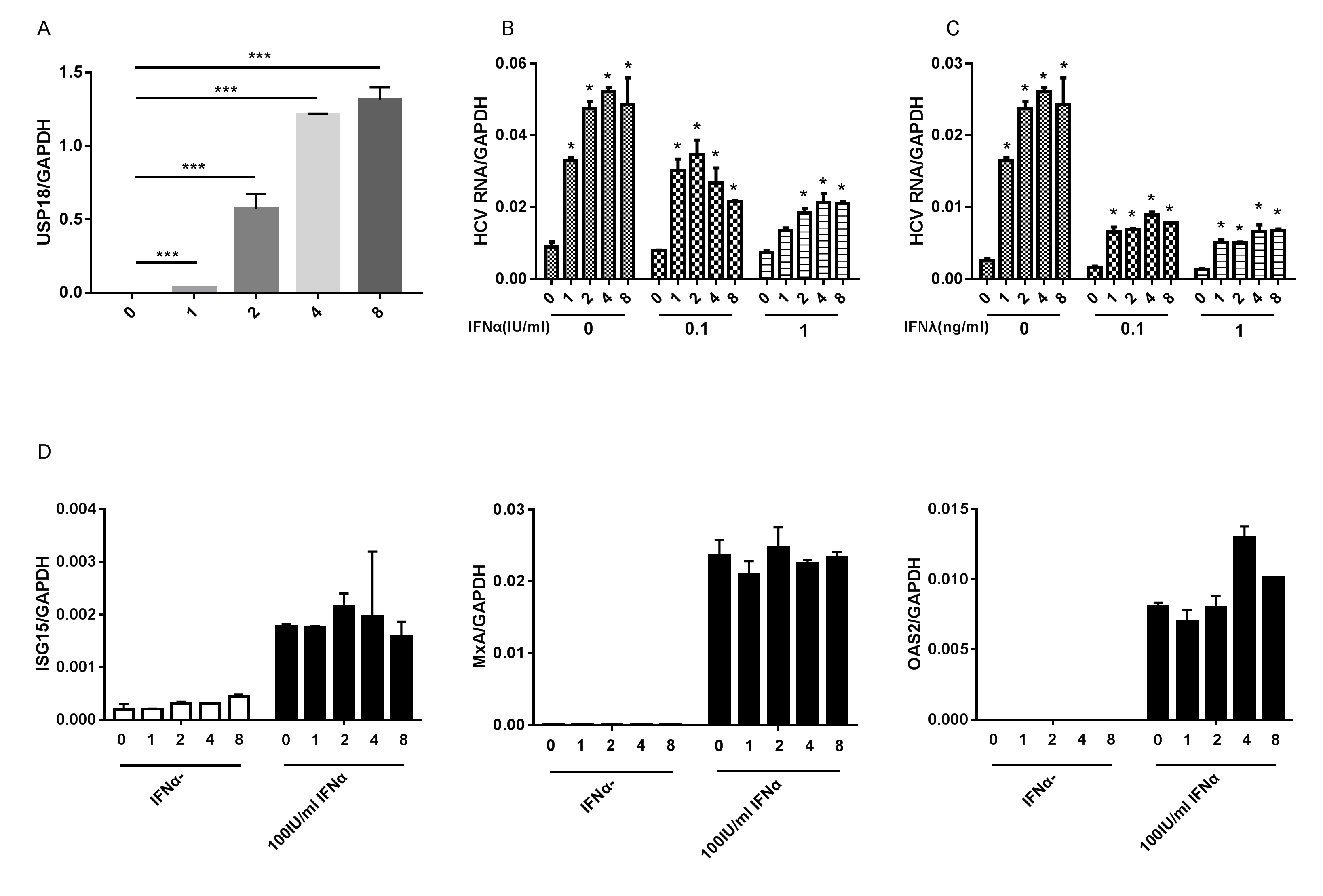
**

**Supplement Figure 3**

**
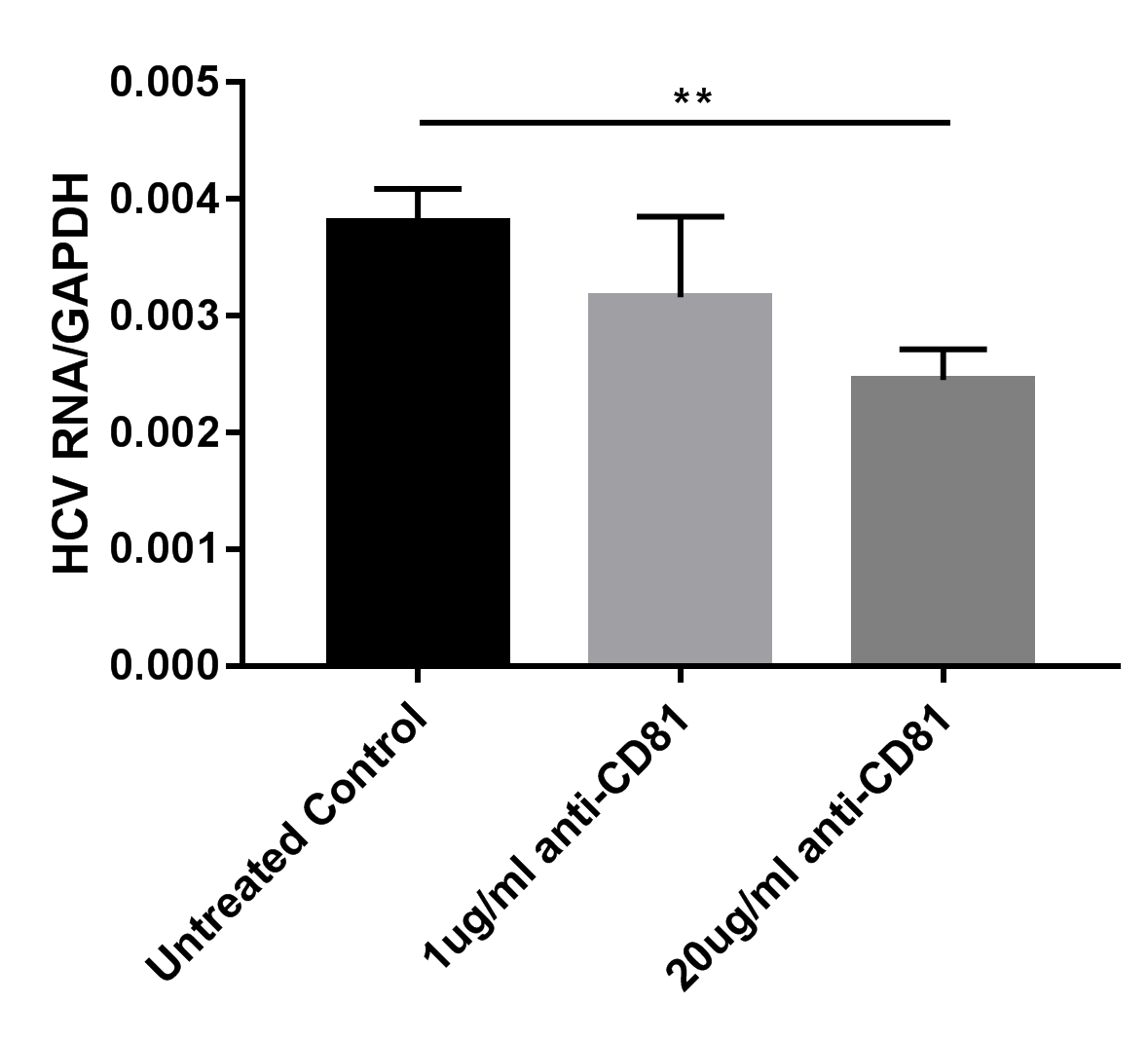
**


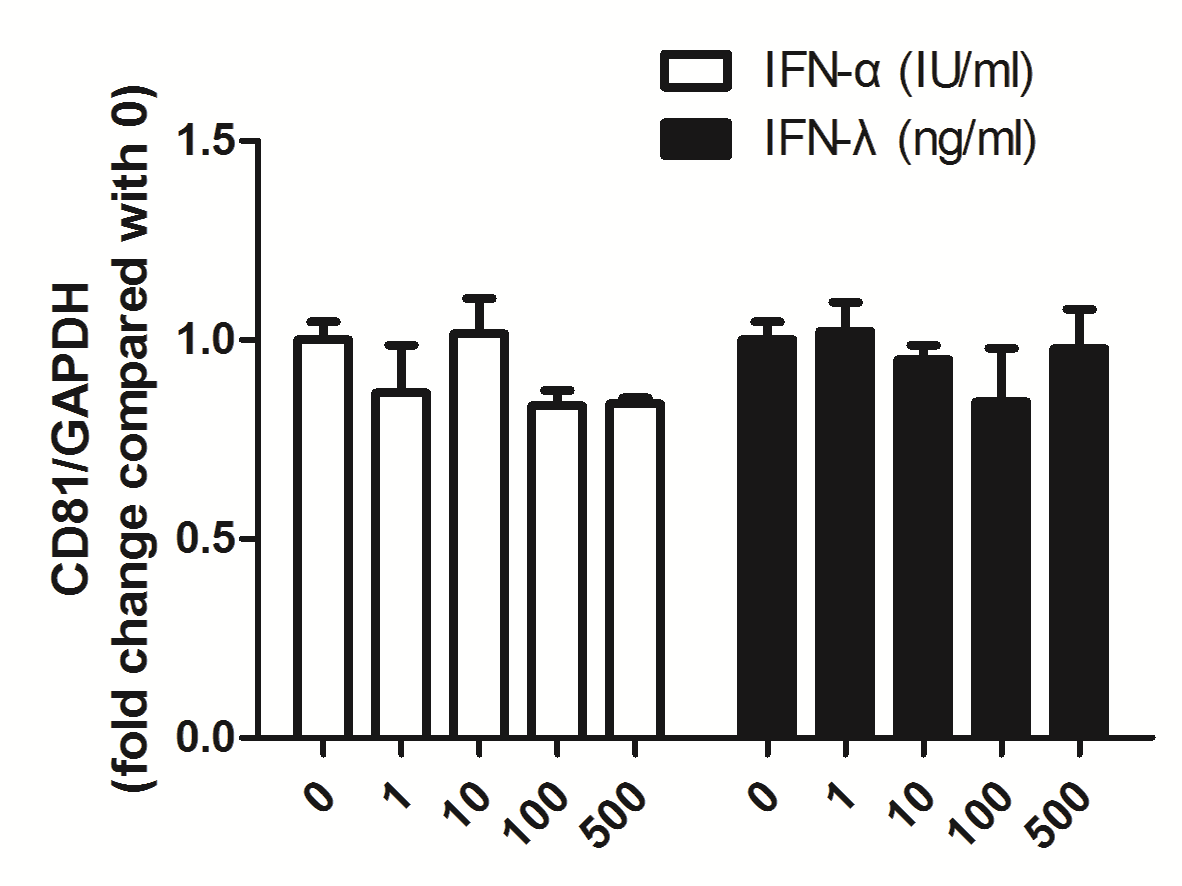
**Supplement Figure 4**

**Supplement Figure 5**

**
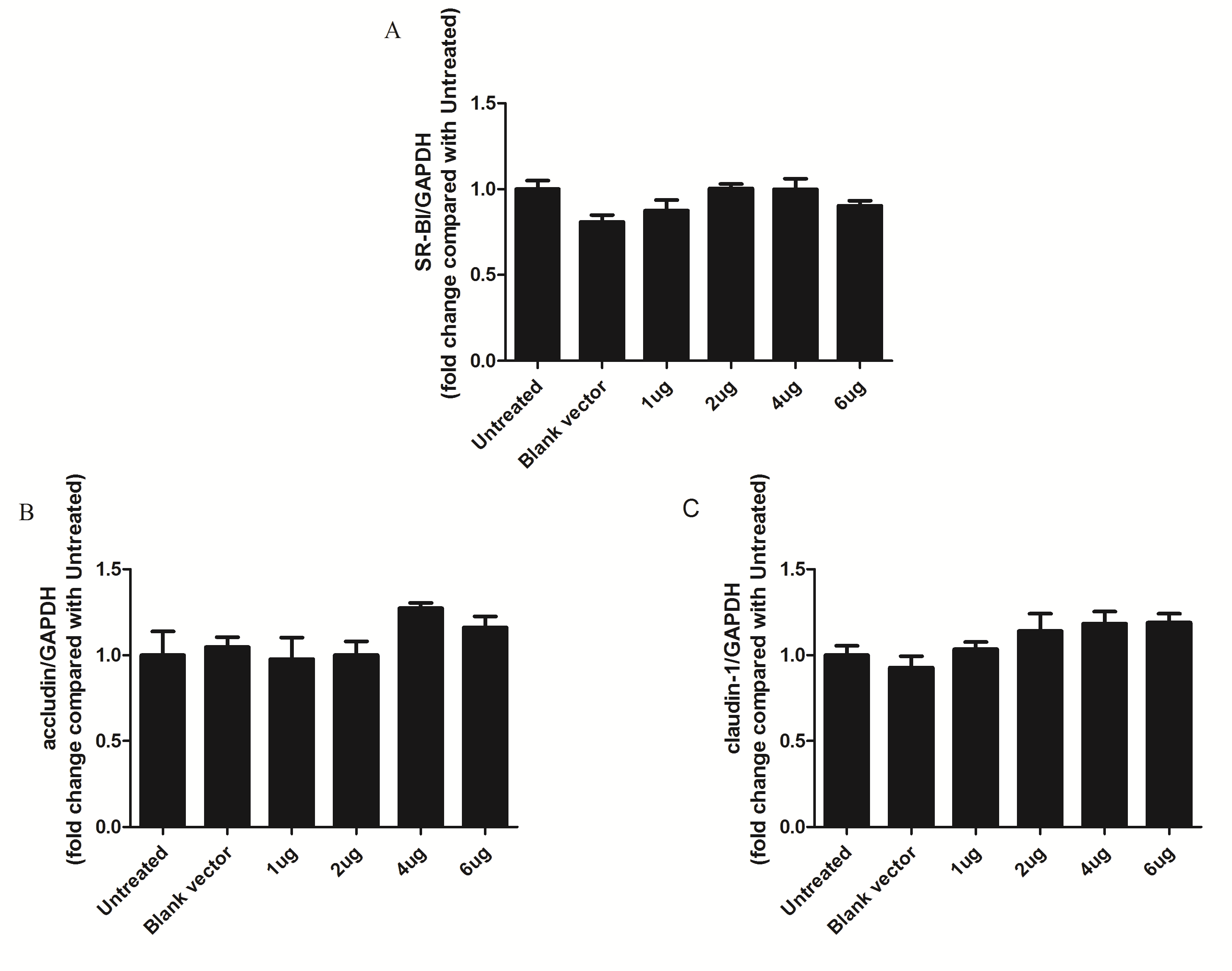
**

**Supplement Figure 1. Transfection efficiency Huh7.5 cells.** Huh7.5 cells were seeded at 3x10^5^/ml, 2ml per well in 6-well plates in antibiotic-free medium for 24 hours before 1ug(**A**), 2ug(**B**), 4ug(**C**) GFP plasmid DNA or 4ug empty vector(**D**) was transfected into each well. Fluorescent microscopy images were taken 48 hours post transfection.

**Supplement Figure 2. Effect of different USP18 plasmid concentrations on IFN signaling pathway and its anti-HCV activity.** Huh7.5 cells were seeded at 3x10^5^/ml, 2ml per well in 6-well plates in antibiotic-free medium for 24 hours before 4ug blank vector pcDNA-DEST53, 1ug, 2ug, 4ug or 8ug USP18 WT was transfected into each well. 48 hours post-transfection, J6/JFH1 virus was added (MOI=4) and incubated for 4 hours before the culture medium was removed. Then the cells were washed and supplied with fresh medium. 8 hours later, IFNα or IFNλ was added at indicated concentrations and the cells was cultured for another 24 hours. The intracellular total RNA was collected. USP18 expression (**A**) without IFN treatment, J6/JFH1 RNA treated with IFNα(**B, left**) or IFNλ(**B, right**) or ISG expression(**C**) with IFNα were detected by real-time PCR, respectively. 0, transfected with 4ug blank vector pcDNA-DEST53; 1, 2, 4 or 8, transfected with 1ug, 2ug, 4 ug or 8ug USP18 WT. Results are presented as means ± SD. *, p < 0.05 compared with group 0.

**Supplement Figure 3. Blocking CD81 decreased intracellular J6/JFH1 HCV RNA.** Huh7.5 cells were seeded at 3x10^5^/ml, 2ml per well in 6-well plates in antibiotic-free medium for 24 hours before one hour preincubation with 1µg/ml or 20µg /ml anti-CD81. Then the cells were washed 3 times by PBS before the J6/JFH1 virus was added (MOI=4). After 4 hours of incubation, the cells were washed and left 24hours before collecting total intracellular RNA. J6/JFH RNA was detected by real-time PCR and normalized to GAPDH. Results are presented as means ± SD. **, p < 0.01 compared with untreated control.

**Supplement Figure 4. Expression of CD81 in USP18-transfected cells in combination with different IFN concentrations and types.** Huh7.5 cells were seeded at 3x10^5^/ml, 2ml per well in 6-well plates in antibiotic-free medium for 24 hours before 4ug USP18 WT was transfected into each well. 48 hours post transfection, IFNα or IFNλ was added into the culture medium at indicated concentrations. 24 hours post IFN treatment, total RNA was extracted, USP18 and CD81 expression was quantified by qRT-PCR, and normalized to GAPDH expression as previously described.

**Supplement Figure 5. Effect of USP18 on other entry or attachment factors.** Huh7.5 cells were seeded at 3x10^5^/ml, 2ml per well in 6-well plates in antibiotic-free medium for 24 hours before 1ug, 2ug, 4ug, 6ug USP18 WT or 4ug empty vector pcDNA-DEST53was transfected into each well. 48 hours post transfection, total RNA was extracted, SR-BI(**A**), occludin(**B**) and claudin-1(**C**) were quantified by qRT-PCR and normalized to GAPDH expression as previously described. Results are presented as means ± SD.
